# Arsenic in Oxyphosphates Not Injurious

**Published:** 1900-04-15

**Authors:** W. V. B. Ames

**Affiliations:** Chicago, Ills.


### ARSENIC IN OXYPHOSPHATES NOT INJURIOUS.

BY DR. W. V. B. AMES, CHICAGO, ILLS.

In view of the extent of the agitation of the question of the trace of arsenic often contained in cement powder, being a potent factor in the occasional unexplained death of a tooth pulp, I consider it advisable to endeavor to sift that matter down.

I do not think that it will be amiss to venture what may be information to some, that this trace of arsenic, in whatever form it may be, is derived from the original zinc ore, which almost invariably has an arsenical contamination. In the distillation of an arsenical zinc ore, the arsenic will be the first component to pass over into the condensing chambers, and if a manufacturer of dental cement happens to get his zinc or oxide from the early portions of a run, then his finished product will almost necessarily have a trace of some compound of arsenic, but if he happens to secure a quantity from the proper stage in the run, he has —generally unknown to himself—a product free from arsenic. In the results of the various series of tests of cement powders for arsenic, I am satisfied that it was a "fluke" in most cases in which a powder has been found to be arsenic free. In tests of powder of my manufacture, some specimens have been found arsenic free and some found to contain a trace, without any more care having been expended in the preparation in one case than the other. In the report recently published by Dr. J. E. Hinkins of tests of powders of different makes, one color of "Lynton" was found free of arsenic, while another contained a trace. I am in position to be pretty sure that this difference did not come from the pigmenting process.

The obtaining of arsenic free raw material is a discouraging undertaking, and if the usual process of producing chemically pure zinc oxide be adopted, the trace of alkaline precipitant which is so persistently present, will be more objectionable than is the trace of the arsenic compound.

In a paper read before the National Dental Association, August, 1899, I gave in detail experiments which proved conclusively to my mind that the arsenic present in cement powder is present as arsenite of zinc, and other experiments, which proved still more conclusively that arsenite of zinc, *per se*, can not bring about the death of a tooth pulp. In the paper referred to I did not deny the possibility of arsenous oxide being developed under some unpardonable conditions. Some recent opinions, from recognized authorities, lead me to believe that I was too conservative in that paper, and that neither the type of phosphoric acid forming the active ingredient of cement liquid nor any putrefactive or fermentative change of organic tissue can develop a potent arsenic compound from arsenite of zinc.

It seems unfortunate that this question should have been brought out in such a way as to constitute a "scare-crow" to young practitioners who have not had sufficient experience to enable them to arrive at an opinion of their own as to its importance. Since it must have come up sooner or later, if it can be thoroughly and effectually sifted at this time all will be well, and some credit redound to some of those instrumental in setting up the "scare-crow."

After what I have offered, it is almost needless for me to say that I do not believe that the statement of Vernon J. Hall in "Chemistry and Matallurgy Applied to Dentistry" in regard to cement powders containing *arsenious oxide*, can be substantiated.

But pulps do often die in a mysterious manner, it is said, under oxyphosphate. If it were from arsenic present in some form in the oxyphosphate, depth of cavity would make little difference, as all know who have made even moderate use of arsenious acid for devitalizing purposes.

If the arsenic feature be eliminated there are left, as I view it, two possible factors, shock and irritation produced by the phosphoric acid.

When a tooth with a vital pulp has been denuded of its enamel, and a portion of its dentin has been cut away in preparing it for a cap crown, and pulp inflammation more or less acute ensues, the symptoms are not those of arsenical irritation, which will hurriedly cut off the circulation of blood within the pulp, but are akin to those symptoms caused at all times by shock of thermal changes, which, if sufficiently severe, will start pulp calcification, that very prevalent source of neuralgias. The death of the pulp of a tooth which has been denuded of its enamel can be accounted for without considering the possible effect which might be exerted by the phosphoric acid under some conditions.

To get down to the question of whether the phosphoric acic may or may not act as a sufficient irritant to cause the death of a tooth pulp we must look into the chemistry of this acid. Theoretically and commercially it is supposed to exist in three forms, meta, pyro and ortho-phosphoric, which may be best regarded as consisting of phosphoric anhydride with one, two and three molecules of water respectively. Meta phosphoric acid is capable of coagulating gelatin and egg albumen. The pyro and ortho-phosphoric are not coagulants of these materials. A sample of acid may really be a combination of meta and pyro phosphoric or pyro and ortho phosphoric depending on the temperature to which the material has been subjected. Glacial phosphoric acid of commerce is meta-phosphoric acid in combination with meta-phosphate of sodium, added for its glacial consistency. A solution of this material in water subjected to moderate heat for a considerable time will give ortho phosphoric acid and ortho phosphate of sodium. A very large proportion of cement used is made up with a solution of this kind, and it is not reasonable to suppose that such a combination can be especially irritant if possessed of reasonable setting qualities. I can only see that there might possibly be danger of irritation from some very slow-setting cement if placed quite near to a tooth pulp, for instance, much less than a millimeter removed. Some of the heavy liquids which give very slow-setting probably border on being of the pyro-phosphoric type. One cement, the "Harvard," is labeled "Pyro-Meta-Phosphatische," etc. I feel called on to say simply that the nearer this liquid comes to being what its label implies the worse it is for the tooth pulp with which it is brought in close proximity. A pyro-phosphate solution would be a heavy liquid, giving slow setting and free acid decidedly in evidence. A pyro meta-phosphate solution would have the same qualities in a more marked degree, and in addition would be a coagulant of albumen. I do not believe that the liquid of this cement is as bad as the label would indicate.

I am inclined to believe that, given a tooth with a cavity approaching very near to the pulp, the chances of saving that pulp in its vital state are much greater if a layer of some bland material such as gutta-percha or a resin in solution is placed over the region of near exposure, than if the oxyphosphate be used exclusively, especially if this be of a slow-setting variety with free acid plainly in evidence. Personally, I prefer oxychloride for covering all nearly exposed pulps, with generally in addition a film of gutta-percha over the region most nearly exposed.

In conclusion, I will say what I expect to find variously said in your discussion, that the death of pulps beneath oxyphosphate fillings and cappings are numerous with some practitioners, because very questionable conditions are so often covered up with this material. In my own practice with a free use of inlays, set with oxyphosphate, for ten years, in all sorts of cavities and a generous use of cement for filling in certain conditions for twenty years, there has been no suggestion or indication of oxyphosphate being a destroyer of vitality of tooth pulps. If my own experiments and those of others were not sufficient to give me a conviction, my clinical experience would justify me in continuing my free use of oxyphosphates.—*Items of Interest*, *February*, 1900.

